# Evaluation of the role of HLA-DR antigens in Japanese type 1 autoimmune hepatitis

**DOI:** 10.1186/s12876-015-0360-9

**Published:** 2015-10-21

**Authors:** Yohei Furumoto, Toru Asano, Tomonori Sugita, Hiroshi Abe, Yoshimichi Chuganji, Kazuhiko Fujiki, Akihiko Sakata, Yoshio Aizawa

**Affiliations:** 1 Department of Internal Medicine, Tokyo Metropolitan Bokutoh Hospital, 4-23-15 Kotobashi, Sumida, Tokyo Japan; 2 Department of Gastroenterology and Hepatology, the Jikei University School of Medicine Katsushika Medical Center, Tokyo, Japan; 3 Department of Pathology, the Jikei University School of Medicine Katsushika Medical Center, Tokyo, Japan

**Keywords:** Autoimmune hepatitis, HLA-DR4, Standard therapy, Treatment response, Japanese, Diagnosis

## Abstract

**Background:**

The role of HLA-DR antigens in the clinicopathological features of autoimmune hepatitis (AIH) is not clearly understood. We examined the implications of HLA-DR antigens in Japanese AIH, including the effect of HLA-DR4 on the age and pattern of AIH onset, clinicopathological features, and treatment efficacy.

**Methods:**

A total of 132 AIH patients consecutively diagnosed and treated in 2000–2014 at 2 major hepatology centers of eastern Tokyo district were the subjects of this study. The frequency of HLA-DR phenotypes was compared with that in the healthy Japanese population. AIH patients were divided into HLA-DR4–positive or HLA-DR4–negative groups and further sub-classified into elderly and young-to-middle-aged groups, and differences in clinical and histological features were examined. Clinical features associated with the response to immunosuppressive therapy were also determined.

**Results:**

The frequency of the HLA-DR4 phenotype was significantly higher in AIH than in control subjects (59.7 % vs. 41.8 %, *P* < 0.001), and the relative risk was 2.14 (95 % CI; 1.51–3.04). HLA-DR4–positive AIH patients were younger than HLA-DR4–negative patients (*P* = 0.034). Serum IgG and IgM levels were higher (*P* < 0.001 and *P* = 0.007, respectively) in HLA-DR4–positive patients. These differences were more prominent in elderly AIH patients. However, there was no difference in IgG and IgM levels between HLA-DR4–positive and HLA-DR4–negative patients of the young-to-middle-aged group. There were no differences in the histological features. In patients with refractory to immunosuppressive therapy, higher total bilirubin, longer prothrombin time, lower serum albumin, and lower platelet count were found. Imaging revealed splenomegaly to be more frequent in refractory patients than in non-refractory patients (60.0 % vs. 30.8 %, *P* = 0.038). HLA-DR phenotype distribution was similar regardless of response to immunosuppressive therapy.

**Conclusions:**

HLA-DR4 was the only DR antigen significantly associated with Japanese AIH. The clinical features of HLA-DR4–positive AIH differed between elderly patients and young-to-middle-aged patients. Treatment response depended on the severity of liver dysfunction but not on HLA-DR antigens.

## Background

Autoimmune hepatitis (AIH) is a rare inflammatory liver disease, with prevalence rates of 5–20 per 100,000 in Europe and North America [1, 2]. Although the etiology of AIH remains unknown, AIH predominantly affects women and is characterized by a marked elevation of serum immunoglobulin levels and the emergence of autoantibodies [3, 4]. The diagnosis relies on a combination of indicative features of AIH and exclusion of other liver diseases. To confirm the diagnosis of AIH, a set of diagnostic criteria, the International Diagnostic Criteria for the Diagnosis of AIH, is generally applied [5]. AIH is classified as type 1 or type 2 according to the type of autoantibodies [6–8]. In Japan, most cases of AIH have been found to be of type 1 [9].

As regards the immunogenetic background of AIH, HLA-DR3 (recently split into DR17 and DR18) and HLA-DR4 are associated with type 1 AIH [10]. In Japan, HLA-DR4 is frequently found in AIH patients, as has been shown in European or North American Caucasoid patients. However, HLA-DR3–positive AIH is quite rare, because the prevalence of DR3 is extremely rare in the normal Japanese population [9].

In a report on North American patients, the clinical features of HLA-DR4–positive AIH differed from those of HLA-DR4–negative patients [11]. In addition, the clinical features of AIH in elderly patients differed from those of younger patients [12–14]. Recently, a lower frequency of HLA-DR4 and a higher frequency of histologically acute hepatitis were reported in adolescent and early adulthood AIH [14]. Moreover, elderly AIH has been increasing in Japan. However, the role of the HLA-DR antigen on the clinical features, including age at onset of AIH and treatment efficacy, has not been extensively studied.

In the present study, we thoroughly examined the role of HLA-DR antigens in Japanese AIH, including how HLA-DR4 influences the age of AIH onset and its clinical features. The association of HLA-DR antigens with the treatment efficacy was also examined.

## Methods

### Study population and study design

A total of 132 patients who had been consecutively diagnosed with AIH, treated, and examined for the HLA-DR antigen at Tokyo Metropolitan Bokutoh Hospital and the Jikei University School of Medicine Katsushika Medical Center (2 of the major hepatology centers in eastern Tokyo district) from the beginning of 2000 till May 2014 were the subjects of this study. AIH diagnosis was based on the Diagnostic Criteria of the International Autoimmune Hepatitis Group (IAHG) [2], which defines AIH on the basis of definite or empirical judgment by experienced hepatologists after ruling out other liver disease such as primary biliary cirrhosis, drug-induced liver disease, hemochromatosis, primary sclerosing cholangitis, Wilson’s disease, α1-antitrypsin deficiency, active cytomegalovirus infection, active Epstein–Barr virus infection, non-alcoholic steatohepatitis, congestive liver injury, and ischemia.

The medical records of the subjects at the time of diagnosis were collected and analyzed retrospectively. In addition, the clinical course of all AIH patients was surveyed. The collected laboratory data included aspartate aminotransferase (AST), alanine aminotransferase (ALT), alkaline phosphatase (ALP), total bilirubin (TB), albumin (Alb), platelet count (PLT), prothrombin time (PT), immunoglobulin G (IgG), immunoglobulin M (IgM), anti-nuclear antibodies (ANA), anti-mitochondrial antibodies (AMA), HCV- and HBV-related viral markers, drug history, average alcohol intake, liver histology, other concomitant autoimmune diseases, and other defined autoantibodies. ANA and AMA were detected and titrated by standard indirect immunofluorescence. For imaging, computed tomography and/or ultrasonography were performed for all but 4 patients, and the presence or absence of splenomegaly was evaluated by radiologists. HLA-DR antigens were assayed in all patients by PCR-based reverse sequence specific oligonucleotide typing (SRL or BML, Tokyo, Japan). Pre-treatment AIH score was calculated in every patient. Then, the frequencies of HLA-DR phenotypes were assessed, and the importance of HLA-DR4 in clinical features in elderly or younger AIH patients was analyzed. The effect of HLA-DR antigens on response to immunosuppressive therapy was also examined.

The protocol of this study was approved by the Ethical committee of Tokyo Metropolitan Bokutoh Hospital and the Ethical committee of the Jikei University School of Medicine. Information of the protocol of this study was explained to the participants, and verbal informed consent was obtained from all patients. This study complied with the standards of the 2008 Declaration of Helsinki and current ethical guidelines.

### Sub-grouping of AIH patients according to HLA-DR4

AIH patients were divided into HLA-DR4–positive and HLA-DR4–negative groups. They were further sub-classified into an elderly group (diagnosed at ≥65 years; 49 patients) and a young-to-middle-aged group (diagnosed at ≤55 years; 49 patients). In each age group, the differences in the clinical features between HLA-DR4 − positive and HLA-DR4 − negative patients were examined.

### Histological examination

Sufficient liver tissue (longer than 2 cm, obtained by 16- or 18-gauge needles) was obtained by percutaneous liver biopsy before starting therapy in 116 out of 132 AIH patients. Among the remaining 16 patients, consent for liver biopsy could not be obtained in 2; immunosuppressive drugs had already been taken based on the clinical diagnosis of AIH in 3; and percutaneous liver biopsy was contraindicated because of severe liver damage in 8 patients. Biopsy specimens could not be obtained in 2 patients, and sufficient length of biopsy specimen was not achieved in 1 patient. The biopsy samples were stained by hematoxylin/eosin and Masson’s trichrome or Azan. The pathological features of AIH were determined collectively by a single pathologist (AS) blinded to clinical information. Pathological scoring of AIH based on the degree of interface hepatitis, predominance of lymphoplasmacytic infiltrate, and presence of rosetting of liver cells was performed according to the diagnostic criteria of the IAHG [2]. In addition, histological staging was assessed based on the Metavir score (F0: no fibrosis, F1: portal fibrosis without septa, F2: portal fibrosis with few septa, F3: numerous septa without cirrhosis and F4: cirrhosis). F0-F2 was considered non-to-mild fibrosis, while F3-F4 was considered advanced fibrosis.

### Criteria of acute or chronic AIH

Patients with acute liver dysfunction (serum ALT levels higher than 10 times of the upper normal limit) or with acute liver-related symptoms (fatigue, jaundice, and appetite loss) without evidence of liver dysfunction in the past (more than 6 months before diagnosis) was defined as acute-onset AIH, while the others were defined as chronic onset AIH. This classification was based on a report by Miyake et al. [15].

### Distribution of HLA-DR antigens in AIH

HLA-DR phenotype frequencies in AIH were determined and compared with those in a large-scale population study on healthy Japanese people by the HLA Laboratory, Kyoto, Japan [16].

### Treatment of AIH patients

Of 132 AIH patients, 121 (92 %) were treated by prednisolone alone or in combination with an immunomodulator (azathioprine, cyclosporine, or tacrolimus). The remaining 11 patients were treated with ursodeoxycholic acid alone because of mild inflammatory activity [17]. Prednisolone alone and in combination with azathioprine was defined as the “standard therapy.” The initial doses of prednisolone were 0.2–1.0 mg/kg except for 2 patients for whom methylprednisolone pulse therapy (1000 mg/day) was selected. Prednisolone doses were gradually decreased to maintenance doses of 10 mg/day or less. Azathioprine doses were adjusted to 0.5–1 mg/kg and maintained [2]. The other immunomodulators were used for patients who were resistant to the standard therapy or could not continue azathioprine use because of adverse reaction [2, 18, 19].

### Statistical analysis

Results are expressed as number (%) or median (minimum–maximum). Fisher’s exact test or chi-square test was used to analyze differences in categorical data. The Mann–Whitney *U*-test was used to analyze differences between continuous variables. Statistical significance was determined by a two-tailed test and *P*-values of ≤0.05 were considered significant. All statistical analyses were carried out using STATISTICA for Windows version 6 (StatSoft, Tulsa, OK, USA).

## Results

### Frequency of HLA-DR phenotypes in AIH patients

The frequency of the HLA-DR4 phenotype was significantly higher in AIH than in control individuals (59.7 % vs. 41.8 %, P < 0.001). The relative risk (RR) of HLA-DR4 was 2.14 (95 % CI; 1.51–3.04).

Among the other HLA-DR antigens, HLA-DR14 tended to be more frequent and HLA-DR11, HLA-DR12, and HLA-DR15 less frequent in AIH compared with control subjects, although the differences were not statistically significant (Table 1).

**Table 1:** Frequency of HLA–DR phenotypes in Japanese patients with autoimmune hepatitis

HLA–DR phenotype	Autoimmune hepatitis (*n* = 132)	Control subjects (*n* = 31973)	*P*–value	Odds ratio (95 % Cl)
1	16 (11.9 %)	3645 (11.4 %)	0.795	1.07 (0.63–1.81)
**4**	**80 (59.7 %)**	**13365 (41.8 %)**	**<0.001**	**2.14 (1.51–3.04)**
7	1 (0.7 %)	239 (0.75 %)	0.622	1.01 (0.14–7.28)
8	38 (28.4 %)	7513 (23.5 %)	0.153	1.32 (0.90–1.92)
9	27 (20.1 %)	8505 (26.6 %)	0.111	0.71 (0.46–1.08)
11	2 (1.5 %)	1644 (5.143 %)	0.092	0.28 (0.07–1.15)
12	8 (6.0 %)	3485 (10.9 %)	0.100	0.53 (0.26–1.08)
13	14 (10.4 %)	4029 (12.6 %)	0.577	0.82 (0.44–1.43)
14	25 (18.7 %)	4348 (13.6 %)	0.074	1.48 (0.96–2.30)
15	34 (25.4 %)	10647 (33.3 %)	0.066	0.69 (0.47–1.03)
16	3 (2.2 %)	575 (1.8 %)	0.935	1.27 (0.40–4.00)
17	1 (0.7 %)	88 (0.28 %)	0.824	2.77 (0.38–20.0)
6 ^*1^	3 (2.2 %)	–		

*^1^ Defined as DR6 before DR6 was further split into DR13 and DR14

Bold-faced type shows statistically significant difference

### Comparison of clinical features between HLA-DR4–positive and HLA-DR4–negative AIH

HLA-DR4–positive AIH patients were younger than HLA-DR4–negative patients. Serum IgG and IgM levels were higher in HLA-DR4–positive patients than in HLA-DR4–negative patients. Splenomegaly was less frequently seen in HLA-DR4–positive AIH, but the difference was not statistically significant. Otherwise, differences were not observed in the remaining demographic or laboratory data, including AST, ALT, ALP, ANA titers, fibrosis stage or pretreatment AIH scores (Table 2).

**Table 2:** Clinical features of HLA–DR4–positiveand HLA–DR4–negativeautoimmune hepatitis

	HLA–DR4–positive AIH (*n* = 78)	HLA-DR4-negative AIH (*n* = 54)	*P*–value
Discrete traits	N (%)	N (%)	
Sex			0.524
Male	8 (10.3)	3 (5.6)	
Female	70 (89.7)	51 (94.4)	
Pattern of disease onset Acute onset	27 (34.6)	20 (37.0)	0.854
Other autoimmune diseases	16 (20.5)	7 (13.0)	0.352
Death from liver disease–related causes	3 (3.8)	5 (9.3)	0.271
ANA positivity	71 (91.0)	44 (81.5)	0.121
ANA (<40/40/80/>80)	7/9/18/44	10/8/10/26	0.435
AMA positivity	9 (11.5)	4 (7.4)	0.558
Splenomegaly	21 (27.6)	23 (44.2)	0.090
Fibrosis stage (F0–F2/F3–F4)	38/31	31/16	0.255
Continuous traits	Median (Min–Max)	Median (Min–Max)	*P*–value
**Age (years)**	**56 (15–86)**	**65 (9–88)**	**0.034**
Total bilirubin (mg/dl)	1.1 (0.4–20.4)	1.1 (0.5–33.2)	0.957
Aspartate aminotransferase (U/L)	203.5 (21–2738)	254.5 (20–1536)	0.930
Alanine aminotransferase (U/L)	270 (15–2325)	284 (16–2016)	0.779
Alkaline phosphatase (U/L)	448.5 (213–1145)	459 (131–1463)	0.860
Gamma–glutamyl transpeptidase (IU/L)	145.5(26–1404)	147.5 (20–679)	0.842
Prothrombin time (%)	86 (29–130)	82 (3–130)	0.251
Albumin (g/dL)	3.7 (1.9–4.7)	3.8 (2.2–4.7)	0.239
Platelets (x 10^4^/nL)	17.7 (8.9–39.6)	17.55 (6.8–31.5)	0.185
**Immunoglobulin G (mg/dL)**	**2582.5 (938–6750)**	**2074.5 (945–4961)**	**<0.001**
**Immunoglobulin M (mg/dL)**	**181 (62–1720)**	**115 (47–888)**	**0.007**
AIH score (except for histology score)	14 (6–18)	13 (7–17)	0.117
AIH score (including histology score)	18 (9–22)	17 (8–22)	0.450

Bold-faced type shows statistically significant difference

### Differences in the effect of HLA-DR4 between young-to-middle-aged and elderly AIH patients

**Fig. 1:**
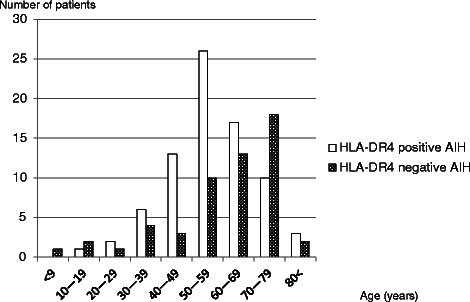
Age distribution of HLA-DR4–positive and DR4-negative autoimmune hepatitis (AIH) patients. Open bars show the number of DR4-positive AIH patients. Dotted bars show the number of DR4-negative AIH patients

According to the age distribution of HLA-DR4–positive and HLA-DR4–negative AIH patients, AIH developed most frequently in individuals in their 50s in HLA-DR4–positive patients and in their 70s in HLA-DR4–negative patients. HLA-DR4–positive AIH was more frequent than HLA-DR4–negative AIH in the age group of 20–59 years. HLA-DR4–negative AIH, however, was more frequent than HLA-DR4–positive AIH in individuals in their 70s (Fig. 1)

In AIH patients diagnosed at age 65 and above (elderly AIH), 22 patients had HLA-DR4, while 27 did not. Both serum IgG and IgM levels were higher in elderly HLA-DR4–positive AIH patients than in HLA-DR4–negative patients. These differences between HLA-DR4–positive and HLA-DR4–negative elderly AIH patients were greater than those shown with the whole population. Moreover, pretreatment AIH scores (including histological score) were significantly higher in the HLA-DR4–positive group than in the HLA-DR4–negative group. Advanced fibrosis tended to be more frequent in HLA-DR4–positive elderly AIH patients (Table 3). With regard to the HLA-DR distribution in elderly AIH patients, the HLA-DR4 phenotype comprised only 44.9 %, and none of HLA-DR antigens were associated with elderly AIH.

**Table 3:** Clinical features of HLA-DR4-positiveand HLA-DR4-negativeautoimmune hepatitis in elderly patients (>65 years old)

	HLA-DR4-positive AlH (*n* = 22)	HLA-DR4-negative AIH (*n* = 27)	*P*-value
Discrete traits	N (%)	N (%)	
Sex			0.084
Male	3 (13.6)	0 (0)	
Female	19 (86.4)	27 (100)	
Pattern of disease onset Acute onset	4 (18.2)	11 (40.7)	0.123
Other autoimmune diseases	4 (18.2)	2 (7.4)	0.388
Death from liver disease-related causes	2 (9.10	3 (11.1)	1.000
ANA positivity	22 (100)	24 (88.9)	0.242
ANA (<40/40/80/<80)	0/1/6/15	3/5/6/13	0.065
AMA positivity	5 (22.7)	2 (7.4)	0.219
Splenomegaly	9 (40.9)	8 (30.8)	0.548
Fibrosis stage (F0-F2/F3-F4)	6/11	16/7	0.053
Continuous traits	Median (Min-Max)	Median (Min-Max)	*P*-value
Age (years)	72 (65–86)	73 (66–88)	0.657
Total bilirubin (mg/dL)	1.1 (0.5–19.0)	1.1 (0.5–33.2)	0.976
Aspartate aminotransferase (U/L)	188 (58–1177)	290 (20–641)	0.376
Alanine aminotransferase (U/L)	191 (62–1208)	284 (16–714)	0.330
Alkaline phosphatase (U/L)	441.5 (239–1145)	443 (170–793)	0.802
Gamma-glutamyl transpeptidase (IU/L)	130.5 (47–466)	203 (20–587)	0.287
Prothrombin time (%)	80.5 (30–104.8)	82 (49–114.4)	0.481
Albumin (g/dL)	3.5 (2.2–4.5)	3.8 (2.2–4.6)	0.190
Platelets (x 10^4^/pL)	14.85 (9.6–27.7)	15.7 (8.0–28.8)	0.920
**Immunoglobulin G (mg/dL)**	**2724.5 (1758–5495)**	**2030 (1243–3157)**	**<0.0001**
**Immunoglobulin M (mg/dL)**	**215 (62–1330)**	**98 (47–396)**	**0.003**
AIH score (except for histology score)	14 (7–17)	13 (7–16)	0.053
**AIH score (including histology score)**	**19 (12–22)**	**17 (8–21)**	**0.037**

Bold-faced type shows statistically significant difference

In AIH patients younger than 55 years (young-to-middle-aged AIH), 34 of 49 (63.8 %) patients had HLA-DR4. The clinicopathological features between HLA-DR4–positive and HLA-DR4–negative AIH were similar except for splenomegaly, which was less frequently found in HLA-DR4–positive patients. In contrast to elderly AIH, serum levels of IgG and IgM or pretreatment AIH scores (including histological score) did not differ between HLA-DR4–positive and HLA-DR4–negative patients. Moreover, the fibrosis stage was similar between HLA-DR4–positive and HLA-DR4–negative AIH (Table 4).

**Table 4:** Clinical features of HLA-DR4-positiveand HLA-DR4-negativeautoimmune hepatitis in young-to middle-aged patients (<55 years old)

	HLA-DR4-positive AIH (*n* = 34)	HLA-DR4-negative AIH (*n* = 15)	*P*-value
Discrete traits	N (%)	N (%)	
Sex			1.000
Male	5 (14.7)	2 (13.3)	
Female	29 (85.3)	13 (86.7)	
Pattern of disease onset Acute onset	17 (50.0)	7 (46.7)	1.000
Other autoimmune diseases	7 (20.6)	4 (26.7)	0.717
Death from liver disease-related causes	1 (2.9)	0 (0)	1.000
ANA positivity	28 (82.4)	10 (66.7)	0.275
ANA (<40/40/80/>80)	7/5/9/13	5/2/2/6	0.642
AMA positivity	4 (11.8)	1 (6.7)	1.000
**Splenomegaly**	**6 (18.8)**	**10 (71.4)**	**0.002**
Fibrosis stage (F0-F2/F3-F4)	23/8	8/5	0.478
Continuous traits	Median (Min-Max)	Median (Min-Max)	*P*-value
Age (years)	46.5 (15–54)	36 (9–54)	0.178
Total bilirubin (mg/dL)	1.1 (0.4–30.4)	1.1 (0.6–18.6)	0.965
Aspartate aminotransferase (U/L)	313.5 (21–2314)	213 (42–1536)	0.957
Alanine aminotransferase (U/L)	499.5 (15–2207)	252 (39–2016)	0.983
Alkaline phosphatase (U/L)	477 (245–732)	491 (184–1463)	0.357
Gamma-glutamyl transpeptidase (IU/L)	181 (26–1404)	166 (23–679)	0.558
Prothrombin time (%)	94 (29–130)	85 (47–130)	0.389
Albumin (g/dL)	4.0 (2.2–4.7)	4.1 (2.9–4.7)	0.267
Platelets (x 10^4^/pL)	21.65 (9.7–39.6)	18.0 (8.5–31.5)	0.175
Immunoglobulin G (mg/dL)	2116.5 (938–6750)	2305 (945–4961)	0.374
Immunoglobulin M (mg/dL)	179.5 (65–890)	231 (59–888)	0.428
AIH score (except for histology score)	12 (6–16)	13 (8–17)	0.197
AIH score (including histology score)	16 (9–21)	17 (9–22)	0.112

Bold-faced type shows statistically significant difference

### Treatment outcome and HLA-DR antigens

Normalization of serum ALT/AST level was accomplished within 6 months in 106 out of 121 patients by the standard therapy. In the remaining 15 patients, 8 developed liver failure and/or died due to liver-related complications, and 7 went into remission by additional dosage of cyclosporine or tacrolimus.

The acute-onset AIH was more frequent in the patients in whom remission was induced by the standard therapy within 6 months (non-refractory group) than in the patients resistant to the standard therapy (refractory group). Splenomegaly was less frequently found in the non-refractory group than in the refractory group. TB was lower, PT was longer, and Alb and PLT were higher in the non-refractory group. Moreover, the serum ALP level was lower in the non-refractory group. The occurrence of advanced liver fibrosis was not different, but the number of patients who did not undergo liver biopsy was higher in the refractory group than in the non-refractory group (40.0 % vs. 8.5 %, *P* = 0.003). In the refractory group, the 6 patients who did not undergo liver biopsy were those for whom liver biopsy was contraindicated because of severe hepatic deterioration (Table 5). HLA-DR phenotype distribution was similar between the non-refractory and refractory groups.

**Table 5:** Clinical features of refractory and non-refractory groups

	Non-refractory group (*n* = 106)	Refractory group (*n* = 15)	*P*-value
Discrete traits	N (%)	N (%)	
Sex			0.356
Male	11 (10.4)	0 (0)	
Female	95 (89.6)	15 (100)	
**Pattern of disease onset**	**45 (42.5)**	**2 (13.3)**	**0.045**
Acute onset			
Other autoimmune diseases	19 (17.9)	3 (20.0)	0.735
**Death from liver disease-related causes**	**0 (0)**	**8 (53.3)**	**<0.001**
ANA positivity	92 (86.8)	13 (86.7)	1.000
ANA (<40/40/80/>80)	14/15/18/59	2/2/6/5	0.274
AMA positivity	13 (12.3)	0 (0)	0.366
**Splenomegaly**	**32 (30.8)**	**9 (60.0)**	**0.038**
Fibrosis stage (F0-F2/F3-F4)	55/42	5/4	1.000
Continuous traits	Median (Min-Max)	Median (Min-Max)	*P*-value
Age (years)	57 (9–87)	64 (46–85)	0.167
**Total bilirubin (mg/dL)**	**1.1 (0.4–30.4)**	**5.1 (0.8–33.2)**	**0.001**
Aspartate aminotransferase (U/L)	248 (21–2738)	133 (46–1130)	0.229
Alanine aminotransferase (U/L)	310 (15–2325)	284 (30–741)	0.429
**Alkaline phosphatase (U/L)**	**456 (131–1463)**	**564 (420–830)**	**0.007**
Gamma-glutamyl transpeptidase (IU/L)	161 (26–679)	192 (48–1404)	0.532
**Prothrombin time (%)**	**86 (29–130)**	**73 (43–109.1)**	**0.002**
**Albumin (g/dL)**	**3.8 (1.9–4.7)**	**3.2 (2.7–4.0)**	**0.004**
**Platelets (x 10**^**4**^**/μL)**	**18.0 (8.9–39.6)**	**11.1 (6.8–36.8)**	**0.016**
Immunoglobulin G (mg/dL)	2536 (938–6750)	2163(1654–5495)	0.514
Immunoglobulin M (mg/dL)	135.5 (47–1720)	172.5 (52–321)	0.618
AIH score (except for histology score)	14 (6–18)	13 (9–16)	0.748
AIH score (including histology score)	18 (8–22)	17 (9–21)	0.284

Bold-faced type shows statistically significant difference

## Discussion

HLA-DR4 is the most important immunogenetic factor responsible for type 1 AIH in Japan [20]. However, the frequency of HLA-DR4 in other countries ranges widely, from 3 % to 59 % [21–25]. In a study of Italian and North American type 1 AIH, HLA-DR4 was found to be less frequent in both Italian type 1 AIH and in control Italian individuals compared with North American counterparts [26]. Although HLA-DR4 is less frequent in Italy, the frequency of HLA-DR4 in the control population of Italy is also less than that of North American counterparts. Although HLA-DR4 was not associated with type1 AIH in Italy, the RR of HLA-DR4 for type 1 AIH in Italy (1.51) and North America (1.46) is quite similar. Therefore, the variation in the frequency of HLA-DR4 in type 1 AIH in different countries may partly depend on the variation in the frequency of HLA-DR4 in the background populations, and hence there is no global consensus on the association of HLA-DR4 and type 1 AIH.

A study on Japanese type 1 AIH revealed a very high occurrence of HLA-DR4 (>80 %) [27], warranting an extensive re-assessment of the distribution of HLA-DR antigens in Japanese AIH. Therefore, in this study, we evaluated the significance of HLA-DR antigens in Japanese type 1 AIH patients. We found that HLA-DR4 alone was significantly associated with Japanese type 1 AIH, but the frequency was as low as 59.7 %. Meanwhile, HLA-DR4 is the most frequent DR antigen (41.7 %) in the normal Japanese population [16]. The RR of HLA-DR4 for type 1 AIH is 2.14, much higher than that for the Italian/North American-type 1 AIH. Other HLA-DR antigens, including HLA-DR13 and HLA-DR3 (DR17 or DR18) [28–31], were not associated with AIH in our study population.

We next examined the role of HLA-DR4 on the clinical features of type 1 AIH. HLA-DR4–positive AIH patients were young and had hypergammaglobulinemia without decreases in albumin levels or platelet count. Marked hypergammaglobulinemia might be a common feature of HLA-DR4–positive AIH in Japan [32]. According to age distribution, about 70 % of AIH patients who were <30 years old were reported to be HLA-DR4– negative [32]. In our patients, although the number of patients younger than 30 years old was relatively small, a tendency of decreasing HLA-DR4 in teens and children was observed.

Interestingly, the incidence of HLA-DR4 differed between elderly and young-to-middle-aged patients. The rate of HLA-DR4 in elderly patients was equal to that in normal subjects. Therefore, HLA-DR4 is not necessarily a risk factor contributing to the onset of AIH in elderly individuals. However, in North America, the frequency of DR4-positive AIH has been increasing in elderly patients, with a decline of DR3-positive AIH [12]. Meanwhile, in Japan, a higher frequency of advanced fibrosis without a change in HLA-DR status has been reported in elderly AIH patients [14]. In our study, the increase in advanced fibrosis in elderly AIH patients was limited to HLA-DR4 patients.

A critical difference between the present study and the earlier one might be the high proportion of elderly AIH patients in our study. Only 20 % of the patients were elderly in the past study, while 34.1 % of the patients in the current study were elderly. This can be attributed to the increase in older population in Japan and recent progress in the proper diagnosis of AIH onset in the elderly. In addition, when the results were compared with those of Caucasian type 1 AIH, HLA-DR3–positive AIH was negligible in the Japanese population.

HLA-DR4–positive elderly AIH patients exhibited prominent hyperglobulinemia, which is one of the characteristic features of typical AIH. In contrast, HLA-DR4–negative elderly AIH patients may exhibit atypical clinical results. Therefore, it is necessary to diagnose HLA-DR4–negative elderly AIH patients carefully.

In young-to-middle-aged AIH patients, the rate of HLA-DR4 was higher. The clinical characteristics were not different between HLA-DR4–positive and HLA-DR4–negative AIH. However, the number of AIH patients younger than 30 years was only 7 in our study. Therefore, precise characterization of AIH in young adults and/or children requires further research. For children or young adults, careful diagnosis of AIH by an experienced hepatologist is essential because differential diagnosis from other causes of liver damage, including chronic active Epstein–Barr virus infection, is difficult [33].

Finally, we examined the contribution of the HLA-DR antigen to the response to immunosuppressive therapy. Association of HLA-DR14 with a favorable response to corticosteroid therapy [34], better treatment response of HLA-DR4 than DR3 [11], lower occurrence of relapse after drug withdrawal and higher frequency of sustained remission in HLA-DR13 [30] have been reported. However, we did not find any differences in HLA-DR between refractory AIH and non-refractory AIH.

Patients with refractory AIH had severe deterioration of liver function. Therefore, we assumed that treatment efficacy was largely influenced by the functional deterioration of the liver at the time of starting therapy. Thus, proper diagnosis and therapy without delay are key factors for the successful treatment of AIH. Meanwhile, the effect of HLA-DR on treatment efficacy may be negligible; however, this cannot be conclusive because of the limited patient number in our study.

## Conclusion

The HLA-DR4 antigen alone was associated with Japanese AIH. No other predisposing HLA-DR antigen was recognized in this study. The impact of HLA-DR4 on the features of AIH differed between elderly patients and younger patients. Treatment response was associated with the severity of liver disease but not with HLA-DR antigen. AIH refractory to immunosuppressive therapy was diagnosed in cases of severe liver dysfunction. Early recognition, proper diagnosis, and immediate start of immunosuppressive therapy are essential for favorable treatment outcome.

## Abbreviations

AIH: Autoimmune hepatitis
IAHG: International Autoimmune Hepatitis Group
AST: Aspartate aminotransferase
ALT: Alanine aminotransferase
ALP: Alkaline phosphatase
TB: Total bilirubin
Alb: Albumin
PLT: Platelet count
PT: Prothrombin time
IgG: Immunoglobulin G
IgM: Immunoglobulin M
ANA: Anti-nuclear antibodies
AMA: Anti-mitochondrial antibodies
RR: Relative risk
